# Associations of Changes in Cardiorespiratory Fitness and Symptoms of Anxiety and Depression With Brain Volumes: The HUNT Study

**DOI:** 10.3389/fnbeh.2019.00053

**Published:** 2019-03-26

**Authors:** Ekaterina Zotcheva, Carl W. S. Pintzka, Øyvind Salvesen, Geir Selbæk, Asta K. Håberg, Linda Ernstsen

**Affiliations:** ^1^Department of Public Health and Nursing, Faculty of Medicine and Health Sciences, Norwegian University of Science and Technology, Trondheim, Norway; ^2^Norwegian National Advisory Unit on functional MRI, Department of Radiology and Nuclear Medicine, St. Olav's Hospital, Trondheim, Norway; ^3^Norwegian National Advisory Unit on Ageing and Health, Vestfold Hospital Trust, Tønsberg, Norway; ^4^Faculty of Medicine, University of Oslo, Oslo, Norway; ^5^Center for Old Age Psychiatric Research, Innlandet Hospital Trust, Ottestad, Norway; ^6^Department of Neuromedicine and Movement Science, Faculty of Medicine and Health Sciences, Norwegian University of Science and Technology, Trondheim, Norway

**Keywords:** exercise, psychiatric symptoms, morphometry, magnetic resonance imaging, aging

## Abstract

**Objective:** We investigated the independent and joint associations of changes in estimated cardiorespiratory fitness (eCRF) and symptoms of anxiety and depression with brain volumes in individuals from the general population.

**Method:** 751 participants (52% women, aged 50–67 years) from the Nord-Trøndelag Health Study (HUNT) MRI cohort were included. eCRF obtained from a non-exercise algorithm and symptoms of anxiety and depression were assessed twice; at HUNT2 (1995–97) and HUNT3 (2006–08). Brain MRI was performed shortly after HUNT3. Brain parenchymal fraction (BPF), bilateral hippocampal and total cortical volume were extracted from brain MRI obtained at 1.5T, using FreeSurfer and Statistical Parametric Mapping.

**Results:** Multiple regression revealed that participants whose eCRF increased had larger BPF (β = 0.09, 95% CI 0.02, 0.16) and larger hippocampal volume (β = 0.09, 95% CI 0.03, 0.16) compared to participants whose eCRF remained low. Participants whose eCRF remained high had larger BPF (β = 0.15, 95% CI 0.07, 0.22) and larger cortical volume (β = 0.05, 95% CI 0.01, 0.09). Participants whose anxiety symptoms worsened had smaller BPF (β = −0.09, 95% CI −0.15, −0.02) and cortical volume (β = −0.05, −0.08, −0.01) than participants whose anxiety symptoms remained low. Each ml/kg/min increase in eCRF was associated with larger cortical volume among individuals with worsening of anxiety symptoms (β = 0.13, 95% CI 0.001, 0.27), and larger BPF among individuals whose depressive symptoms improved (β = 0.28, 95% CI 0.02, 0.53).

**Conclusion:** Promoting exercise intended to improve eCRF may be an important public health initiative aimed at maintaining brain health among middle-aged individuals with and without changing psychological symptoms.

## Introduction

It is well established that the volume of the human brain decreases in normal aging. However, accelerated hippocampal and cortical atrophy may be an indicator of the development of mild cognitive impairment or dementia in older adults (Driscoll et al., 2009; Fotuhi et al., 2012; Hartikainen et al., 2012). Given the aging population and the related personal, economic, and social burdens of cognitive impairment, efforts to uncover risk and protective factors for brain structural and functional decline are currently a priority.

Cardiorespiratory fitness (CRF) has been proposed as a factor which may attenuate age-related brain atrophy (Hayes et al., 2013; Erickson et al., 2014). CRF expresses the ability of the body's circulatory and respiratory systems to support oxidative metabolism during sustained physical activity (PA), and can be improved through aerobic exercise (Ross et al., 2016). Studies have shown an association between CRF and larger whole brain (Zhu et al., 2015), hippocampal (Erickson et al., 2009; Szabo et al., 2011), and cortical volumes (Williams et al., 2017). Furthermore, accumulating evidence suggests that CRF is beneficial for cognition in older adults (Wendell et al., 2014; Freudenberger et al., 2016) and has been associated with a reduced risk of dementia (Defina et al., 2013), emphasizing the importance of CRF for healthy brain aging. As assessment of CRF using exercise tests may be challenging (American College of Sports Medicine, 2014), non-exercise algorithms to estimate CRF (eCRF) have been developed, and have been found to correlate favorably with direct measures of CRF (Nes et al., 2011; Jackson et al., 2012; Nauman et al., 2017). In a cross-sectional study, eCRF was associated with cognitive function and hippocampal volume, and these associations did not differ significantly from when applying objectively measured CRF (McAuley et al., 2011). Thus, eCRF may serve as a suitable proxy for the objectively assessed CRF in epidemiological studies with larger populations, where exercise testing may not be feasible (Ross et al., 2016).

Anxiety and depression have been linked to reduced brain volumes (Mah et al., 2016; Zhang et al., 2018), and increased risk of dementia (Kaup et al., 2016; Petkus et al., 2016). As anxiety and depression are common among middle-aged and older adults (Reynolds et al., 2015), understanding the role of these disorders in relation to brain atrophy is important. There is evidence that exercise interventions and higher levels of CRF lower symptoms of anxiety (Williams et al., 2016; Stubbs et al., 2017) and depression (Schuch et al., 2016a,b). However, there is a lack of studies assessing the association of both CRF and anxiety and depression with brain structure. Furthermore, it is important to understand how changes in CRF and anxiety and depression are associated with aging-related brain areas such as the hippocampus and cortex, as this may provide important information for public health interventions.

The aim of the present study was to investigate the independent and joint associations of concurrent changes in eCRF and symptoms of anxiety and depression over a period of 12 years with brain parenchymal fraction (BPF), which is an estimate of structural brain reserves (Vagberg et al., 2017), total hippocampal and total cortical volume in a middle-aged sample drawn from the general population.

## Methods

### Study Population

Data was collected from the Nord-Trøndelag Health Study (HUNT), a large population-based health survey from the Nord-Trøndelag County in Norway (Krokstad et al., 2013). All adult inhabitants of the county were invited to participate in HUNT1 (1984–1986), HUNT2 (1995–1997), and HUNT3 (2006–2008), with overall participation rates of 89.4, 69.5, and 54.1%, respectively. However, in HUNT3 the participation rate in the age group 60–69 years was 71.1% (Thoen and Krokstad, 2011). In the present study, only data from HUNT2 and HUNT3 was used, as questions regarding physical activity, anxiety, and depression were different at HUNT1.

To be included in the HUNT magnetic resonance imaging (MRI) study, a sub-study in HUNT3, participants were required to have participated in HUNT1,−2, and−3, be between 50 and 65 years of age at inclusion, live within 45 min traveling distance from the location of the MRI examination, and not have standard MRI contraindications such as body weight above 150 kg. Of 1,494 invited participants, 1,088 (73%) agreed to participate in the study, and 1,006 (64.5%, 530 women) had successful MRI examinations and were defined as MRI participants. For details on inclusion and characteristics of participants, non-participants, and non-invited see Honningsvag et al. (2012).

This study was approved by the HUNT study board of directors and the Helse Midt-Norge Regional Ethics and Health Research Committee (REK midt). All participants were legally competent adults, and gave their written informed consent.

### MRI Scan Protocol

Brain MRI was performed using a 1.5 T GE Signa HDx 1.5 T MRI scanner, equipped with an eight-channel head coil (GE Healthcare) and software version pre-14.0M4. All participants underwent the same scan protocol, for details see Haberg et al. (2016). The following MRI scans were used in the current study; The Alzheimer's Disease Neuroimaging Initiate volume, which is a T1 weighted volume (TR = 10,156 ms, TE = 4.044 ms, FOV = 240 mm, slice thickness = 1.2 mm, gap 0 mm, matrix 192 × 192, giving an in plane resolution of 0.94 × 0.94 mm) and an axial T2 weighted sequence (TR = 7.84 ms, TE = 95 ms, FOV = 203 mm, slice thickness = 4 mm, gap 1 mm, matrix 512 × 320, giving an inplane resolution of 0.45 × 0.45 mm).

### MRI Data Analysis

MRI data was analyzed with FreeSurfer V5.3.0 (Fischl and Dale, 2000; Fischl et al., 2002; Whelan et al., 2018) for automatic segmentation of total brain volume, total hippocampal volume, and total cortical volume, area, and thickness in accordance with the ENIGMA pipeline, and quality assessed visually (http://enigma.ini.usc.edu/protocols/imaging-protocols/). The sum of left and right hemisphere volume measurements was used.

Intracranial volume (ICV) was estimated based on a combination of T1 and T2 images using an automated version of the reverse brain mask method (Keihaninejad et al., 2010) termed the “automatic reverse brain mask method” by using the “new segment” approach of the SPM8 (http://www.fil.ion.ucl.ac.uk/spm) toolbox, full description in Hansen et al. (2015). BPF was calculated from total brain parenchymal volume divided by ICV (Juengling and Kassubek, 2003), and is presented as percentage of ICV.

### Estimated Cardiorespiratory Fitness

A previously validated non-exercise prediction model (Nauman et al., 2017) based on sex, age, waist circumference (WC), resting heart rate (rHR), and self-reported PA was used to calculate eCRF, expressed as ml oxygen uptake per kg per minute (ml/kg/min). Participants were stratified into two PA groups based on self-reported intensity, duration, and frequency of PA performed weekly during the past year. In the algorithms presented below, PA = 1 if the participant followed the current recommendations of 150 min moderate or 75 min vigorous physical activity per week (Garber et al., 2011), and PA = 0 if not. The sex-specific algorithms used to predict individual eCRF were (Nauman et al., 2017):

$$\begin{array}{l} Women:78.00-\left(0.297*Age\right)-\left(0.270*WC\right)-\left(0.110*rHR\right)\\ \;+\left(2.674^{*}PA\right)\\ Men:105.91-\left(0.334*Age\right)-\left(0.402*WC\right)-\left(0.144*rHR\right)\\ \;+\left(3.102^{*}PA\right) \end{array}$$

To assess change in eCRF, participants were dichotomized into “low” and “high” eCRF groups based on sex- and age- (≤46 or >46 years at HUNT2, ≤59 or >59 years at HUNT3) specific medians of the eCRF distribution. The participants were then stratified into four eCRF change groups: “remained low” (“low” at both HUNT2 and HUNT3), “decreased” (“high” at HUNT2, “low” at HUNT3), “increased” (“low” at HUNT2, “high” at HUNT3), and “remained high” (“high” at both HUNT2 and HUNT3). For greater statistical power, the continuous change variable delta eCRF (ΔeCRF) was calculated by subtracting eCRF at HUNT2 from eCRF at HUNT3 for each participant. Whereas the categorical change variable provides a relative measure of eCRF change dependent on the age- and sex-specific median, the continuous change variable provides data on absolute change in ml/kg/min from HUNT2 to HUNT3.

### Symptoms of Anxiety and Depression

Symptoms of anxiety and depression were assessed using the Hospital Anxiety and Depression Scale (HADS). The HADS is a self-assessment scale developed in 1983 by Zigmond and Snaith (1983), and is a reliable instrument for detecting and assessing symptoms of anxiety and depression in somatic, psychiatric, and primary care patients, and in the general population (Herrmann, 1997; Bjelland et al., 2002). The Norwegian version of the HADS included in HUNT2 and HUNT3 has good psychometric properties and is in close agreement with the original questionnaire developed by Zigmond and Snaith (Mykletun et al., 2001).

The HADS consists of 7 items that cover anxiety symptoms (HADS-A) and 7 items that cover depressive symptoms (HADS-D), giving a total of 14 items. Each subscale item has a 4-point Likert scale ranging from 0 (no symptom) to 3 (highest symptom level), with a maximum score of 21 on each scale indicating the highest symptom load. Participants who had responded to <5 questions on either the HADS-A (HUNT2 *n* = 3, HUNT3 *n* = 114) or HADS-D (HUNT2 *n* = 2, HUNT3 *n* = 114) scale were excluded from the analyses. For participants who had answered 5 or 6 questions (HUNT2: HADS-A *n* = 67, HADS-D *n* = 28; HUNT2: HADS-A *n* = 17, HADS-D *n* = 14), the total score was extrapolated by multiplying the sum by 7/5 or 7/6, respectively. A total score of 8 or above on the HADS-A or HADS-D subscale was used as an indication of clinically relevant anxiety or depression symptoms, respectively (Bjelland et al., 2002).

Based on the cut-off of ≥8 on the HADS-A and HADS-D scale, participants were classified into “low” (< 8) and “high” (≥8) HADS-categories at HUNT2 and HUNT3. Next, four HADS change groups were created: “remained low” (“low” at both HUNT2 and HUNT3), “improved” (“high” at HUNT2, “low” at HUNT3), “worsened” (“low” at HUNT2, “high” at HUNT3), and “remained high” (“high” at both HUNT2 and HUNT3). For greater statistical power, we also calculated delta HADS-A and HADS-D (ΔHADS-A and ΔHADS-D) by subtracting HADS-scores at HUNT2 from HADS-scores at HUNT3. Whereas the categorical HADS change variables provide a relative measure of HADS change as they depend on the cut-off for clinically relevant symptoms of anxiety and depression, the continuous change variables provide data on absolute change in HADS score from HUNT2 to HUNT3.

### Statistical Analyses

Forty-five participants were excluded from further analyses due to brain pathology, and 50 participants were excluded due to failed or incorrect FreeSurfer processing. Further, 160 participants were excluded due to missing data on eCRF or HADS. The final sample comprised 751 participants (390 women) with a mean age of 58.9 years (range 50–67) at the time of the HUNT MRI examination, and with a mean time of 11.8 ± 0.2 years between HUNT2 and HUNT3.

Descriptive statistics were used to assess study sample characteristics for the four categorical change in eCRF groups. Pearson's correlation (*r*) and Cramér's V (ϕ_*c*_) were computed to estimate correlation between changes in eCRF, HADS-A, and HADS-D. We used multiple regression models to assess standardized regression coefficients (β) and 95% confidence intervals (CI) for the associations of changes in eCRF and HADS, both as categorical and continuous variables, with BPF, total hippocampal volume, and total cortical volume. The “remained low” eCRF and “remained low” HADS groups served as reference categories. The role of age and sex in the eCRF prediction models is to refine the estimate of CRF, not to remove the effect of age and sex in the relationships between eCRF and brain volumes. Thus, all models were adjusted for age, sex, education (highest achieved level: primary, upper secondary, or college/university), and smoking (never, former, or current). Models with hippocampal and cortical volumes were also adjusted for ICV. In addition, models with ΔeCRF and ΔHADS were adjusted for eCRF at HUNT2 and HADS score at HUNT2, respectively.

To assess the joint associations of changes in eCRF and HADS, we investigated the association of ΔeCRF with brain volumes, stratified by categorical change in HADS-A and HADS-D. This allowed us to assess the influence of changes in eCRF on brain volumes among different strata of HADS change. Interaction between categorical measures of eCRF and HADS could not be assessed due to low statistical power. Thus, we investigated interaction on an additive scale between ΔeCRF and ΔHADS on brain volumes. In additional analyses, we assessed the independent associations of changes in eCRF and HADS with brain volumes. Here, ΔeCRF, ΔHADS-A, and ΔHADS-D were included in one model, and categorical measures of change in eCRF, HADS-A, and HADS-D were included in another model. Finally, in order to better capture areas in the cortex that may be associated with changes in eCRF and HADS, we performed analyses with cortical thickness and area. Here, General Linear Models (GLMs) were fitted at each vertex across the cortical surface. Individual surface maps were smoothed with a full-width-half-maximum Gaussian kernel of 25 mm, averaged across participants (Fischl and Dale, 2000). Contrast vectors were set to test for the effect of ΔeCRF, ΔHADS-A, and ΔHADS-D yielding continuous cortical maps. All models were adjusted for the same variables as in the main analyses. The two *p*-value maps from left and right hemisphere were combined and thresholded at <5% False Discovery Rate (FDR).

All statistical analyses were performed with IBM SPSS Version 25 for Windows (SPSS Inc, Los Angeles, USA). A *p* < 0.05 was considered statistically significant.

## Results

Study sample characteristics stratified by change in eCRF are shown in Table 1. Across the study population, there was a mean reduction in eCRF from HUNT2 to HUNT3 of 6.37 ± 3.14 ml/kg/min. The prevalence of clinically relevant anxiety symptoms (HADS-A ≥8) was 13.0% at HUNT2 and 11.3% at HUNT3, whereas the prevalence of clinically relevant depressive symptoms (HADS-D ≥8) was 7.5% at HUNT2 and 9.9% at HUNT3. Mean ΔHADS-A was −0.270 ± 2.97 and mean ΔHADS-D was 0.022 ± 2.70. There was no evidence of any meaningful correlation between changes in eCRF and HADS; ΔeCRF and ΔHADS-A, *r* = 0.03, *p* = 0.410, ΔeCRF and ΔHADS-D, *r* = 0.004, *p* = 0.903, categorical eCRF change and HADS-A change, ϕ_*c*_ = 0.06, *p* = 0.497, categorical eCRF change and HADS-D change, ϕ_*c*_ = 0.09, *p* = 0.024. ΔHADS-A and ΔHADS-D were moderately correlated, *r* = 0.51, *p* < 0.001, as were categorical measures of change in HADS-A and HADS-D, ϕ_*c*_ = 0.61, *p* < 0.001. Results from the multiple regression models assessing the associations of changes in eCRF and HADS with BPF, hippocampal and cortical volume are shown in Tables 2,3.

**Table 1:** Characteristics of study population stratified by change in eCRF, relative to sex-, and age-defined median, from HUNT2 to HUNT3 (*n* = 751).

	**Change in eCRF**			
	**Remained low (*n* = 265)**	**Decreased (*n* = 112)**	**Increased (*n* = 110)**	**Remained high (*n* = 264)**
Age at HUNT MRI, mean, SD	60.3 ± 3.88	56.9 ± 3.34	60.8 ± 3.43	57.6 ± 4.15
Women, *n* (%)	137 (51.7)	59 (52.7)	57 (51.8)	137 (51.9)
University/college, *n* (%)	70 (26.4)	36 (32.1)	33 (30.0)	110 (41.7)
Smokers (current), *n* (%)	67 (25.3)	31 (27.7)	29 (26.4)	58 (22.0)
HADS-A change^*^, ***n*** **(%)**				
Remained low	217 (81.9)	86 (76.8)	92 (83.6)	213 (80.7)
Improved	14 (5.3)	12 (10.7)	9 (8.2)	23 (8.7)
Worsened	17 (6.4)	7 (6.3)	3 (2.7)	18 (6.8)
Remained high	17 (6.4)	7 (6.3)	6 (5.5)	10 (3.8)
HADS-D change^*^, ***n*** **(%)**				
Remained low	217 (81.9)	92 (83.0)	100 (90.9)	235 (89.0)
Improved	11 (4.2)	3 (2.7)	6 (5.5)	12 (4.5)
Worsened	25 (9.4)	9 (8.0)	2 (1.8)	14 (5.3)
Remained high	12 (4.5)	7 (6.3)	2 (1.8)	3 (1.1)
HADS-A HUNT2, mean, SD	3.93 ± 3.06	4.35 ± 3.83	3.53 ± 2.93	3.81 ± 2.99
ΔHADS-A^**^, mean, SD	−0.15 ± 2.99	−0.55 ± 3.41	−0.13 ± 2.52	−0.34 ± 2.92
HADS-D HUNT2, mean, SD	3.56 ± 2.94	3.32 ± 3.26	2.92 ± 2.69	2.67 ± 2.64
ΔHADS-D^**^, mean, SD	0.03 ± 2.90	−0.11 ± 3.01	0.01 ± 2.19	0.08 ± 2.56
eCRF HUNT2, mean, SD	38.5 ± 5.23	43.9 ± 4.76	40.5 ± 4.66	45.3 ± 5.41
ΔeCRF^§^, mean, SD	−6.81 ± 2.82	−9.97 ± 2.25	−3.13 ± 2.19	−5.76 ± 2.35

*eCRF, estimated cardiorespiratory fitness; HADS-A, Hospital Anxiety and Depression Scale anxiety subscale; HADS-D, Hospital Anxiety and Depression Scale depression subscale*.

* *Change in HADS from HUNT2 to HUNT3 based on a cut-off at ≥8 on the HADS-A and HADS-D subscales*.

** *Change in HADS calculated by subtracting HADS-scores at HUNT2 from HADS-scores at HUNT3*.

§ *Change in eCRF calculated by subtracting eCRF at HUNT2 from eCRF at HUNT3*.

**Table 2:** Standardized beta coefficients (β) and 95% confidence intervals (CI) for the associations of changes in eCRF and HADS from HUNT2 to HUNT3 on BPF, hippocampal, and cortical volume measured at HUNT3 (*n* = 751).

		**Brain region**					
		**BPF (mean** **=** 69.7%)^*^		**Hippocampal volume (mean** **=** **7,599** μl)^**^		**Cortical volume (mean** **=** **426,764** μl)^**^	
	***n***	**β (95% CI)**	**Adjusted *R*^**2**^**	**β (95% CI)**	**Adjusted *R*^**2**^**	**β (95% CI)**	**Adjusted *R*^**2**^**
**eCRF change**							
Remained low	265	Ref.	0.22	Ref.	0.40	Ref.	0.77
Decreased	112	0.01 (−0.07, 0.08)		0.04 (−0.02, 0.11)		−0.003 (−0.04, 0.04)	
Increased	110	**0.09 (0.02, 0.16)**		**0.09 (0.03, 0.16)**		0.02 (−0.01, 0.06)	
Remained high	264	**0.15 (0.07, 0.22)**		0.05 (−0.02, 0.12)		**0.05 (0.01, 0.09)**	
**HADS-A change**							
Remained low	608	Ref.	0.21	Ref.	0.40	Ref.	0.77
Improved	58	0.01 (−0.05, 0.08)		−0.01 (−0.06, 0.05)		0.001 (−0.03, 0.04)	
Worsened	45	–**0.09 (**–**0.15**, –**0.02)**		−0.06 (−0.11, 0.001)		–**0.05 (**–**0.08**, –**0.01)**	
Remained high	40	−0.01 (−0.08, 0.05)		0.03 (−0.03, 0.09)		−0.01 (−0.04, 0.03)	
**HADS-D change**							
Remained low	645	Ref.	0.21	Ref.	0.39	Ref.	0.77
Improved	32	0.02 (−0.05, 0.08)		−0.01 (−0.07, 0.05)		−0.01 (−0.04, 0.03)	
Worsened	50	−0.05 (−0.11, 0.02)		−0.03 (−0.08, 0.03)		−0.03 (−0.07, 0.01)	
Remained high	24	−0.04 (−0.10, 0.03)		0.01 (−0.05, 0.07)		−0.002 (−0.04, 0.03)	
ΔeCRF^§^	751	**0.16 (0.10, 0.23)**	0.24	**0.09 (0.03, 0.15)**	0.40	**0.05 (0.02, 0.09)**	0.78
ΔHADS-A^§^	751	–**0.07 (**–**0.15**, –**0.002)**	0.21	−0.05 (−0.12, 0.01)	0.40	–**0.04 (**–**0.08**, –**0.003)**	0.77
ΔHADS-D^§^	751	−0.07 (−0.15, 0.001)	0.21	−0.01 (−0.08, 0.05)	0.39	−0.03 (−0.07, 0.01)	0.77

*BPF, brain parenchymal fraction; eCRF, estimated cardiorespiratory fitness; HADS-A, Hospital Anxiety and Depression Scale anxiety subscale; HADS-D, Hospital Anxiety and Depression Scale depression subscale. Bold text indicates statistically significant associations at p < 0.05*.

* *Adjusted for age, sex, education, and smoking*.

** *Adjusted for age, sex, education, smoking, and intracranial volume*.

§ *Additionally adjusted for eCRF, HADS-A, or HADS-D at HUNT2*.

**Table 3:** Standardized beta coefficients (β) and 95% confidence intervals (CI) for the association of ΔeCRF from HUNT2 to HUNT3 with BPF, hippocampal, and cortical volume measured at HUNT3, stratified by change in HADS (*n* = 751).

		**Brain region**					
		**BPF (Mean** **=** 69.7%)^*^		**Hippocampal volume (Mean** **=** **7,599** μl)^**^		**Cortical volume (Mean** **=** **426,764** μl)^**^	
	***n***	**β (95% CI)**	**Adjusted *R*^**2**^**	**β (95% CI)**	**Adjusted *R*^**2**^**	**β (95% CI)**	**Adjusted *R*^**2**^**
**HADS-A change**							
Remained low	608	**0.17 (0.09, 0.24)**	0.24	**0.08 (0.01, 0.14)**	0.38	**0.04 (0.004, 0.08)**	0.78
Improved	58	0.25 (−0.01, 0.51)	0.26	0.09 (−0.13, 0.30)	0.61	0.08 (−0.07, 0.22)	0.79
Worsened	45	0.07 (−0.17, 0.32)	0.38	0.14 (−0.12, 0.40)	0.44	**0.13 (0.001, 0.27)**	0.82
Remained high	40	0.12 (−0.14, 0.37)	0.24	0.10 (−0.16, 0.36)	0.30	0.02 (−0.14, 0.18)	0.69
**HADS-D change**							
Remained low	645	**0.17 (0.10, 0.24)**	0.23	**0.08 (0.02, 0.14)**	0.39	**0.05 (0.01, 0.09)**	0.77
Improved	32	**0.28 (0.02, 0.53)**	0.49	0.09 (−0.34, 0.51)	0.35	0.15 (−0.02, 0.31)	0.83
Worsened	50	−0.03 (−0.27, 0.21)	0.24	0.16 (−0.09, 0.41)	0.53	0.11 (−0.02, 0.23)	0.85
Remained high	24	0.26 (−0.26, 0.78)	0.42	0.29 (−0.17, 0.76)	0.39	−0.14 (−0.38, 0.09)	0.81

*BPF, brain parenchymal fraction; eCRF, estimated cardiorespiratory fitness; HADS-A, Hospital Anxiety and Depression Scale anxiety subscale; HADS-D, Hospital Anxiety and Depression Scale depression subscale. Bold text indicates statistically significant associations at p < 0.05*.

* *Adjusted for age, sex, education, smoking, and eCRF at HUNT2*.

** *Adjusted for age, sex, education, smoking, intracranial volume, and eCRF at HUNT2*.

### Brain Parenchymal Fraction

Participants in the “increased” eCRF group and in the “remained high” eCRF group had larger BPF; β = 0.09 (95% CI 0.02, 0.16) and β = 0.15 (95% CI 0.07, 0.22), respectively, compared to participants in the “remained low” eCRF group. In addition, each ml/kg/min increase in ΔeCRF corresponded to a larger BPF, β = 0.16 (95% CI 0.10, 0.23).

Participants in the “worsened” HADS-A group had smaller BPF, β = −0.09 (95% CI −0.15, −0.02), compared to participants in the “remained low” HADS-A group. Further, each unit increase in ΔHADS-A was associated with a smaller BPF, β = −0.07 (95% CI −0.15, −0.002). ΔHADS-D was associated with smaller BPF, although this result did not reach statistical significance, β = −0.07 (95% CI −0.15, 0.001) (Table 2).

### Hippocampal Volume

Participants in the “increased” eCRF group had a larger hippocampal volume, β = 0.09 (95% CI 0.03, 0.16), compared to participants in the “remained low” eCRF group. Furthermore, each ml/kg/min increase in ΔeCRF was associated with a larger hippocampal volume, β = 0.09 (95% CI 0.03, 0.15).

Neither change in HADS-A nor HADS-D was significantly associated with hippocampal volume (Table 2).

### Total Cortical Volume

Participants in the “remained high” eCRF group had larger cortical volume, β = 0.05 (95% CI 0.01, 0.09), compared to those in the “remained low” eCRF group. In addition, each ml/kg/min increase in ΔeCRF corresponded to a larger cortical volume, β = 0.05 (95% CI 0.02, 0.09).

Participants in the “worsened” HADS-A group had smaller cortical volume compared to those in the “remained low” HADS-A group, β = −0.05 (95% CI −0.08, −0.01). Further, each unit increase in ΔHADS-A was associated with smaller cortical volume, β = −0.04 (95% CI −0.08, −0.003). Change in HADS-D was not significantly associated with cortical volume (Table 2).

### Stratified Analyses

In analyses stratified by categorical HADS change, each ml/kg/min increase in ΔeCRF was associated with larger BPF (β = 0.17, 95% CI 0.09, 0.24), hippocampal (β = 0.08, 95% CI 0.01, 0.14) and cortical volume (β = 0.04, 95% CI 0.004, 0.08) among participants in the “remained low” HADS-A group. In the “worsened” HADS-A group, each ml/kg/min increase in ΔeCRF was associated with larger cortical volume, β = 0.13 (95% CI 0.001, 0.27). In the “remained low” HADS-D group, each ml/kg/min increase in ΔeCRF was associated with larger BPF (β = 0.17, 95% CI 0.10, 0.24), hippocampal (β = 0.08, 95% CI 0.02, 0.14), and cortical volume (β = 0.05, 95% CI 0.01, 0.09). In addition, each ml/kg/min increase in ΔeCRF was associated with larger BPF in the “improved” HADS-D group, β = 0.28 (95% CI 0.02, 0.53) (Table 3).

### Additional Analyses

Analyses assessing independent changes in eCRF, HADS-A, and HADS-D did not alter the associations, although the association between ΔHADS-A and BPF and ΔHADS-A and cortical volume was attenuated and was no longer statistically significant after adding ΔHADS-D, HADS-D at HUNT2, ΔeCRF, and eCRF at HUNT2 to the model (Supplementary Table 1). No interaction effects of ΔeCRF and ΔHADS on brain volumes were observed (all *p*s ≥ 0.188). Finally, no localized associations were observed for cortical thickness or area.

## Discussion

In our study of 751 adults from the general population, we found that increasing or maintaining a high eCRF during middle-age was associated with larger BPF, hippocampal and total cortical volume. Worsening of anxiety symptoms was associated with smaller BPF and total cortical volume. In the stratified analyses, increasing eCRF was associated with larger brain volumes among individuals whose anxiety and depressive symptoms remained low, among individuals with worsening anxiety symptoms, and among those with improving depressive symptoms.

The increased brain volumes observed among individuals whose eCRF increased or remained high is in accordance with previous studies showing larger whole brain (Zhu et al., 2015), hippocampal (Erickson et al., 2009), and cortical (Williams et al., 2017) volume in individuals with higher levels of CRF measured from exercise testing. Whereas, these studies measured CRF at a single time-point, our study provides evidence for the importance of maintaining high eCRF or increasing one's eCRF with regards to brain volumes. We also found that the associations between changes in eCRF and brain volumes were independent of concurrent changes in symptoms of anxiety and depression. This underlines the significance of eCRF for brain volumes in the aging general population regardless of psychological symptoms. Previous studies examining the relationship between CRF measured from exercise testing and brain volumes have typically had smaller sample sizes (Erickson et al., 2009; Williams et al., 2017). In agreement with earlier findings showing associations between non-exercise CRF algorithms and gray matter volume (McAuley et al., 2011; Boots et al., 2015), our study provides additional support for the use of eCRF in larger population-based studies where exercise testing may be costly and time-consuming (American College of Sports Medicine, 2014).

Earlier studies investigating gray matter volume in anxiety disorders have found smaller volumes in the hippocampus, midbrain, thalamus, insula, and superior temporal gyrus among patients with general anxiety disorder (Moon et al., 2014) and smaller volumes in the rostral anterior cingulate gyrus, the dorsal anterior cingulate gyrus, and left middle/superior temporal area among individuals with panic disorder and social anxiety disorder without comorbid major depressive disorder (van Tol et al., 2010). However, these studies did not take into account changes in anxiety severity over time. In addition, findings in individuals with anxiety disorders are not necessarily transferrable to individuals from the general population with unspecified anxiety symptoms. In this study, we found that individuals with worsening anxiety symptoms had smaller BPF and cortical volume. Thus, our study suggests that reduced gray matter volume is not limited to anxiety disorders, but is also present among individuals in the general population with worsening subclinical symptoms of anxiety.

Surprisingly, we did not observe a statistically significant association between changes in depressive symptoms and brain volumes, although we observed a similar reduction in BPF associated with ΔHADS-D as observed for ΔHADS-A (see Table 2). These non-significant findings are in contrast to several studies showing reduced hippocampal volume among individuals with elevated depressive symptoms (Elbejjani et al., 2015; Szymkowicz et al., 2018). High comorbidity of depression and anxiety (Gorman, 1996), supported by the moderate correlations between changes in symptoms of anxiety and depression in our study, is a possible explanation as to why our results did not support earlier findings. However, the association between changes in symptoms of anxiety and brain volumes was not noteworthy attenuated after adding concurrent changes in depressive symptoms to the regression model, suggesting that symptoms of anxiety and depression are differently associated with brain volumes. It is also worth noting that the prevalence of clinically relevant symptoms of depression was lower than the prevalence of clinically relevant symptoms of anxiety in our sample, and was also lower than in the same age group in the general HUNT sample (Stordal et al., 2001). Thus, it is plausible that we may have obtained different results in a sample with a higher prevalence of depressive symptoms.

To our knowledge, no previous study has investigated the joint associations of long-term changes in eCRF and symptoms of anxiety and depression on brain volumes. The results from stratified analyses showed that increased eCRF was associated with larger cortical volume among participants whose symptoms of anxiety worsened and with larger BPF among participants whose symptoms of depression had improved. With few exceptions, eCRF was positively associated with larger brain volumes across all strata of change in HADS-A and HADS-D, even though a number of these associations did not reach statistical significance. Given that anxiety and depressive symptoms are common among older adults (Reynolds et al., 2015), strategies aimed at reducing brain structural decline, and thus associated consequences such as cognitive decline, in these individuals are of uttermost importance. PA, specifically aerobic exercise, is considered to be one of the main modifiable factors associated with CRF (Ross et al., 2016). Hence, our findings suggest that encouraging PA participation aimed at maintaining or increasing eCRF may attenuate accelerated brain atrophy among middle-aged adults with worsening symptoms of anxiety and those with a history of heightened depressive symptoms.

The present study has several strengths. First, the study included a large, validated, population-based sample with a relatively narrow age range and an acceptable participation rate of 71.1% for the age group 60–69 at HUNT3 (Krokstad et al., 2013). Of those invited to participate in HUNT MRI, 73% agreed to participate (Honningsvag et al., 2012). The demographic data for HUNT MRI participants did not differ from that of non-invited and non-participants, except that participants had less cardiovascular risk factors and higher education (Honningsvag et al., 2012). In addition, the prospective observational design of the study made it possible to assess changes in anxiety and depressive symptoms and eCRF over an extended time period.

The main limitation of our study is the lack of longitudinal brain MRI data, making causal inferences about the observed associations between changes in eCRF, symptoms of anxiety and depression, and change in brain volumes impossible. However, there is evidence indicating that both anxiety (Mah et al., 2016) and exercise that increases CRF (Kandola et al., 2016) may have causal effects on brain volume. Selection bias may have been present, as quite many participants did not complete the HADS at HUNT3. Participants who did not complete the HADS at HUNT3 were slightly younger than those who did, but did not differ with regards to HADS scores at HUNT2, eCRF, or brain volumes (data not shown). Further, the HADS measures symptoms of anxiety and depression during the past week. Thus, we did not have data on symptoms occurring between HUNT2 and HUNT3, possibly decreasing the precision of our results. Similarly, the PA question used in the eCRF calculation only captures PA during the past year. Thus, we do not have data on fluctuations in PA levels that may have occurred between HUNT2 and HUNT3. The study may also be limited by subjective measurements of anxiety, depression, and the PA question used in the calculation of eCRF. However, a cross-sectional study investigating the association between eCRF and hippocampal volume found that eCRF significantly predicted hippocampal volume, and that this association did not differ significantly from the association between objectively assessed CRF and hippocampal volume (McAuley et al., 2011). The non-exercise CRF algorithm used in this study has been found to have similar accuracy and be highly comparable to previously published eCRF algorithms (Nauman et al., 2017). Although the authors went through a number of possible confounding variables and selected the variables included in the regression models carefully, the possibility of residual confounding cannot be completely ruled out, as is the case in most epidemiological studies. In addition, there may be residual confounding due to unmeasured variables, such as prescription medication or cognitive function, which may have influenced both changes in eCRF or HADS and brain volumes.

In conclusion, our study demonstrated that increasing or maintaining high eCRF during midlife was associated with larger BPF, and hippocampal and cortical volume in a sample of middle-aged adults drawn from the general population. Worsening of anxiety symptoms was associated with smaller BPF and cortical volume. Importantly, increased eCRF appeared to be especially beneficial for cortical volume among individuals with worsening anxiety symptoms. In sum, our findings underline the importance of physical activity promotion, and anxiety prevention, as a means of promoting healthy brain aging in the general population. Health care professionals in primary clinical practice should promote physical activity and emphasize treating anxiety symptoms in order to maintain brain health among middle-aged individuals.

## Data Availability

The data analyzed in this study was obtained from the HUNT Research Center. Instructions and requirements for data access are available on the following website https://www.ntnu.edu/hunt/data.

## Author Contributions

EZ, CP, ØS, GS, AH, and LE all contributed to the conceptualization and design of the study, drafting and revising of the manuscript prior to submission and during revision.

### Conflict of Interest Statement

The authors declare that the research was conducted in the absence of any commercial or financial relationships that could be construed as a potential conflict of interest.

## Abbreviations

BPF: brain parenchymal fraction
CRF: cardiorespiratory fitness
eCRF: estimated cardiorespiratory fitness
HADS: Hospital Anxiety and Depression Scale
HADS-A: Hospital Anxiety and Depression Scale anxiety subscale
HADS-D: Hospital Anxiety and Depression Scale depression subscale
HUNT: Nord-Trøndelag Health Study
ICV: intracranial volume
PA, physical activity, rHR: resting heart rate
WC: waist circumference.
