# Supplementary material for: Associations of Changes in Cardiorespiratory Fitness and Symptoms of Anxiety and Depression With Brain Volumes: The HUNT Study
Source: Front Behav Neurosci. 2019 Mar 26;13:53. doi: 10.3389/fnbeh.2019.00053 (PMC6443896; doi:10.3389/fnbeh.2019.00053)
Supplement: Supplementary file 1 [file Table_1.docx]

Supplementary Material

Associations of Changes in Cardiorespiratory Fitness and Symptoms of Anxiety and Depression with Brain Volumes: The HUNT Study

Ekaterina Zotcheva, Carl W. S. Pintzka, Øyvind Salvesen, Geir Selbæk, Asta K. Håberg, Linda Ernstsen*

***Correspondence:**

Linda Ernstsen
Postal address: NTNU, Fakultet for medisin og helsevitenskap, Institutt for samfunnsmedisin og sykepleie, Postboks 8905, N-7491 Trondheim, Norway
Phone no.: +47 73413036
Email: linda.ernstsen@ntnu.no

Supplementary Table 1. Standardized beta coefficients (β) and 95% confidence intervals (CI) for the independent associations of changes in eCRF and HADS from HUNT2 to HUNT3 with BPF, hippocampal volume, and cortical volume (n=751).

|  |  | Brain region | | | | | |
| --- | --- | --- | --- | --- | --- | --- | --- |
|  |  | BPF (Mean = 69.7%)* | | Hippocampal volume (Mean = 7599μl)** | | Cortical volume (Mean = 426764μl)** | |
|  | *n* | β (95% CI) | Adjusted R^2^ | β (95% CI) | Adjusted R^2^ | β (95% CI) | Adjusted R^2^ |
| eCRF change^‡^ |  |  |  |  |  |  |  |
| Remained low | 265 | Ref. | 0.23 | Ref. | 0.40 | Ref. | 0.78 |
| Decreased | 112 | 0.01 (-0.07, 0.08) |  | 0.04 (-0.02, 0.11) |  | -0.004 (-0.04, 0.04) |  |
| Increased | 110 | **0.09 (0.02, 0.16)** |  | **0.09 (0.03, 0.15)** |  | 0.02 (-0.02, 0.06) |  |
| Remained high | 264 | **0.15 (0.07, 0.22)** |  | 0.06 (-0.01, 0.12) |  | **0.05 (0.12, 0.09)** |  |
|  |  |  |  |  |  |  |  |
| HADS-A change^‡‡^ |  |  |  |  |  |  |  |
| Remained favorable | 608 | Ref. | 0.23 | Ref. | 0.40 | Ref. | 0.78 |
| Improved | 58 | 0.01 (-0.06, 0.07) |  | -0.01 (-0.07, 0.05) |  | 0.002 (-0.03, 0.04) |  |
| Worsened | 45 | **-0.08 (-0.15, -0.01)** |  | -0.05 (-0.11, 0.01) |  | **-0.04 (-0.08, -0.01)** |  |
| Remained unfavorable | 40 | -0.003 (-0.07, 0.07) |  | 0.03 (-0.03, 0.09) |  | -0.01 (-0.04, 0.03) |  |
|  |  |  |  |  |  |  |  |
| HADS-D change^‡‡‡^ |  |  |  |  |  |  |  |
| Remained favorable | 645 | Ref. | 0.23 | Ref. | 0.40 | Ref. | 0.78 |
| Improved | 32 | 0.01 (-0.06, 0.08) |  | -0.02 (-0.07, 0.05) |  | -0.01 (-0.05, 0.03) |  |
| Worsened | 50 | -0.003 (-0.07, 0.07) |  | -0.001 (-0.06, 0.06) |  | -0.01 (-0.05, 0.03) |  |
| Remained unfavorable | 24 | -0.02 (-0.08, 0.05) |  | 0.01 (-0.05, 0.07) |  | 0.01 (-0.03, 0.05) |  |
|  |  |  |  |  |  |  |  |
| ΔeCRF^§^ | 751 | **0.16 (0.10, 0.23)** | 0.24 | **0.09 (0.03, 0.15)** | 0.40 | **0.06 (0.02, 0.09)** | 0.78 |
| ΔHADS-A^§§^ | 751 | -0.06 (-0.14, 0.02) | 0.24 | -0.07 (-0.14, 0.01) | 0.40 | -0.05 (-0.09, 0.001) | 0.78 |
| ΔHADS-D^§§§^ | 751 | -0.03 (-0.12, 0.06) | 0.24 | 0.03 (-0.05, 0.11) | 0.40 | 0.004 (-0.04, 0.05) | 0.78 |

BPF: brain parenchymal fraction; eCRF: estimated cardiorespiratory fitness; HADS-A: Hospital Anxiety and Depression Scale anxiety subscale; HADS-D: Hospital Anxiety and Depression Scale depression subscale. Bold text indicates statistically significant associations at p<0.05.
*Adjusted for age, sex, education, and smoking.
**Adjusted for age, sex, education, smoking, and intracranial volume.
^§^Additionally adjusted for eCRF, HADS-A, and HADS-D at HUNT2, ΔHADS-A, and ΔHADS-D.

^§§^Additionally adjusted for eCRF, HADS-A, and HADS-D at HUNT2, ΔeCRF, and ΔHADS-D.

^§§§^Additionally adjusted for eCRF, HADS-A, and HADS-D at HUNT2, ΔeCRF, and ΔHADS-A.

^‡^ Additionally adjusted for categorical HADS-A and HADS-D change.

^‡‡^ Additionally adjusted for categorical eCRF and HADS-D change.

^‡‡‡^ Additionally adjusted for categorical eCRF and HADS-A change.
